# A Virtual Reality Cognitive Stimulation Program as an Effective Tool Against Residual/Prodromal Depressive Symptoms in Bipolar Disorders

**DOI:** 10.3390/jcm13164714

**Published:** 2024-08-11

**Authors:** Mauro Giovanni Carta, Peter K. Kurotschka, Sergio Machado, Andreas Erfurth, Federica Sancassiani, Alessandra Perra, Massimo Tusconi, Giulia Cossu, Cesar Ivan Aviles Gonzalez, Diego Primavera

**Affiliations:** 1 Department of Medical Sciences and Public Health, University of Cagliari, 09042 Cagliari, Italy; maurogcarta@gmail.com (M.G.C.); federicasancassiani@yahoo.it (F.S.); alessandra.perra@unica.it (A.P.); giuliaci@hotmail.com (G.C.); infermiere2020@gmail.com (C.I.A.G.); 2 Department of General Practice, University Hospital Würzburg, 97070 Würzburg, Germany; kurotschka_p@ukw.de; 3 Center of Neuroscience, Neurodiversity Institute, Queimados 26325-010, Brazil; secm80@gmail.com; 4 Institute of Psychiatry-IPUB, Federal University of Rio de Janeiro, Rio de Janeiro 22290-140, Brazil; 5 6th Psychiatric Department, Otto-Wagner-Spital, 1140 Vienna, Austria; andreas.erfurth@meduniwien.ac.at; 6 Azienda Ospedaliero Universitaria di Cagliari, 09042 Cagliari, Italy; massimotusconi@yahoo.com

**Keywords:** bipolar disorder, depressive symptoms, cognitive remediation training, virtual reality, advanced technology laboratory, patient health questionnaire

## Abstract

**Background:** Bipolar disorder (BD) is a severe psychiatric illness characterized by a chronic course and recurrent episodes, including residual depressive symptoms even during euthymic phases. These symptoms, although not meeting criteria for a depressive episode, are linked to relapse risk and impaired social functioning. This study aims to assess whether Virtual Reality Cognitive Remediation Training reduces depressive symptoms below the clinical threshold in individuals with BD. **Methods:** This post hoc analysis focuses on the secondary outcome (PHQ9) of a randomized–controlled trial. Participants were recruited from the Center of Liaison Psychiatry and Psychosomatics in Italy. The experimental group received Virtual Reality Cognitive Remediation, while the control group received standard treatment **Results:** Data from 39 individuals in the experimental group and 25 in the control group were analyzed. A greater reduction in PHQ-9 scores (>9) was observed in the experimental group (71.8% to 48.7%) compared to the control group. Significant improvements in total PHQ-9 scores and specific symptoms were noted in the experimental group compared to the control group. **Conclusions:** The study highlights the significant impact of virtual reality intervention on reducing depressive symptoms in bipolar disorder. This promising outcome underscores the potential preventive role of cognitive stimulation in relapse prevention. The intervention could offer valuable benefits for both treatment and prevention strategies in bipolar disorder.

## 1. Introduction

Bipolar disorder (BD) is a serious psychiatric disorder and one of the leading causes of disability, making it a globally relevant health issue [1,2,3,4]. BD impacts the life of people suffering from it due the higher rate of comorbidity and the associated risk of suicide [5,6]. The prevalence in the community is around 2–4% according to different surveys conducted worldwide [3,7,8]. The onset of the disorder is frequently in adolescence or early adulthood, but BD has a chronic course often lasting over a lifetime, with possible recurrences, each of which worsens the course and residual functionality [9,10]. In bipolar disorder, even during the so-called euthymic phases, residual depressive symptoms have been found with a frequency of approximately 50–70% [11,12]. These subsyndromal manifestations, which do not meet the required criteria for defining a depressive episode, are closely associated with the risk of relapse and low social functioning [13]. Indeed, it has been observed that the presence of residual depressive symptoms primarily influences adherence to pharmacological treatment, a critical element in bipolar disorder. The outcome of this treatment is strongly affected by the regularity of therapy adherence [14,15]. Subthreshold depressive symptoms are also typical during stress phases when individuals with traits of hyperthymia and novelty-seeking may experience the onset of bipolar disorders [16,17,18]. These considerations highlight the importance of therapeutic tools that can impact subsyndromal depressive symptoms in individuals with bipolar disorder or at risk of developing it. The results of a recently conducted randomized–controlled clinical trial on the efficacy of Virtual Reality Cognitive Remediation Training (NCT05070065), aimed at improving the cognitive performance of individuals with bipolar disorder in the euthymic phase, demonstrated a greater reduction in depressive symptoms (measured by the PHQ-9 score [19,20]) in the experimental group compared to the control group [21]. Virtual reality (VR) offers several advantages over traditional paper-and-pencil tasks when designing effective cognitive training programs. To highlight a few, VR boasts enhanced ecological validity, meaning there is a greater resemblance between the training environment and the real world, which is believed to add value in predicting improvements in daily functioning. Secondly, a VR environment—like any digital test—provides the opportunity to offer immediate feedback on performance, which is widely acknowledged as essential for most learning processes and successful rehabilitation. Lastly, VR allows for the customization of environments and activities, making them more engaging [22].

### Aims

The aim of this study was to measure if the improvement, namely in decreasing by time the number of people with the number of symptoms, is over the threshold level for a depressive episode. It is in fact known that the threshold defined by PHQ9 can be as reliable as a screener if associated with functional impairment; that is, it is not enough to be positive and have a certain number of symptoms, but to have a diagnosis of depression, a low level of functionality is necessary. In our sample, there are people with acceptable functionality and therefore not in a critical phase; however, there is such a large number of symptoms (as often happens in the stability phases of bipolar disorder) that the screener would identify them as “positive” (i.e., >9). We wanted to see, through a secondary analysis on an outcome defined as secondary in the research project, namely the PHQ9 score, to what extent the reduction was in order to bring people back even below the screener threshold, i.e., clinical significance. It was also our goal to understand the specific symptoms on which virtual reality has a greater effect.

## 2. Materials and Methods

### 2.1. Design of the Study

This survey is a post hoc analysis focused on a secondary outcome (PHQ9) of a randomized–controlled trial, registered in ClinicalTrials.gov (NCT05070065, 2021). The project was carried out in agreement with the CONSORT extension guidelines [23]. 

### 2.2. Sample, Setting, and Randomization

The sample included people with a diagnosis of bipolar disorder at time of recruitment, in absence of current manic/depressive episode, and receiving treatment at the Care Unit of the Center of Liaison Psychiatry and Psychosomatics of the “Ospedale Civile San Giovanni di Dio” in Cagliari, Italy (University Hospital of Cagliari). Users could be included in the study if they were aged between 18 and 75 years and had received a diagnosis of bipolar disorder according to DSM-IV criteria [24] from a clinical psychiatrist. There was no exclusion based on gender or ethnicity. In addition to people presenting with a current manic/depressive episode, people with serious eye diseases or epilepsy were also excluded because for these disorders, risks were detected in the administration of virtual reality [25,26]. Each selected participant had to sign an informed consent before the beginning of the survey. The researchers that conducted evaluations at T0 and T1 were blinded of the intervention the proband under evaluation underwent (experimental or control).

### 2.3. Experimental and Control Intervention

A fully immersive Virtual Reality Cognitive Remediation program was administered to the experimental group. A description of the adopted software “CEREBRUM” was presented in a previously published paper [21]. The software was implemented by “PRoMIND” and “IDEGO”, two small/medium-sized Italian high-tech enterprises. The rehabilitation strategy was designed in such a way as to subject the users to tasks of gradually increasing difficulty. The supervising clinician was given the opportunity to adapt the difficulty of the intervention in relation to the participants’ performance and to adapt the task to the user’s specific abilities or potentially improvable gaps. This strategy also had the aim of making the route stimulating and less boring. The experimental group was subjected to 24 sessions with virtual reality of approximately 45 min each, with a frequency of two sessions per week; the duration of the overall intervention was three months. The package was conceived through a human-centered approach [27] that was recovery- and social inclusion-oriented [28,29]. The two compared groups (experimental and control) received treatment as usual (i.e., psychiatric treatment with clinical interviews or pharmacotherapy with the possibility of receiving, if deemed by the psychiatric specialist, psychoeducational interventions or psychotherapy). Therefore, the study compared the experimental group that underwent virtual reality plus treatment as usual to the control group, which underwent only treatment as usual.

### 2.4. Outcome and Measures

This paper is focused on the measure of depressive symptoms that was planned as a secondary outcome in the deposited protocol [21]. The outcome measure is the score obtained on the Patient Health Questionnaire—9-item version (PHQ9) [20,30], adopted in the Italian validated version [21]. The total score of PHQ scale is obtained by the sum of the score of each item. The nine items each inquire about one of the core symptoms of the depressive episode according to the DSM-5 (Diagnostic and Statistical Manual of Mental Disorders) [31]. According to Kroenke’s work [19], a score lower than 5 almost always identifies the absence of a depressive disorder; scores of 5 to 9 identify people without depression and with subthreshold symptoms, while the important clinical threshold was 10 because having an equal or higher score was frequently associated with clinically relevant depression. Our sample is a sample of people diagnosed with bipolar disorder but without current crises. It therefore seemed important to us to verify whether the treatment resulted in an improvement below 10 in a significant number of people compared to the control group.

The improvement in each item (core symptom of the DSM diagnosis) was calculated as a comparison in the difference of improvement of the average scores on that single PHQ9 item (T0 vs. T1) in the experimental group and in the control group.

### 2.5. Statistical Analysis

The comparison in the change in number of persons with more than 9 points in the over score of PHQ9 in the two groups by time (T0 vs. T1) was calculated by means of analysis of variance by nominal data by Castella [32], which uses chi-square statistics.

The difference within groups at each timepoint (T1 vs. T0) for the PHQ total and for each PHQ item score was calculated as the difference in the mean score ± standard deviation by means ANOVA 1-way statistics, and a *p*-value < 0.05 was considered statistically significant.

## 3. Results

As described in the previous survey on the main outcome, the experimental sample consisted of 39 individuals. Of the 50 recruited, 11 did not complete the intervention and dropped out at follow-up. The control group comprised 25 individuals with no dropouts. No statistically significant differences were found between the experimental and control groups in terms of mean age (47.51 ± 13.52 in the experimental group vs. 46.28 ± 13.40 in the control group; F (1,63) = 0.127; *p* = 0.723) or in the frequency of sex (females 78.9% in the experimental group vs. 80% in the control group; chi-square (1) = 0.43; *p* = 0.51).

In the experimental group, individuals with a PHQ9 score greater than 9 decreased from 28 (71.8%) at T0 to 19 (48.7%) at T1 (chi-square = 4.33, *p* = 0.03). In the control group, individuals with a PHQ9 score greater than 9 remained steady at 10 (40%) from T0 to T1 (chi-square with Yates correction = 0, *p* = 1). Table 1 shows the change over time in individuals with a PHQ9 score greater than 9 in both groups, where the experimental group saw a 23.1% improvement compared to 0% in the control group (Castellan chi-square = 6.713, *p* = 0.01).

Table 2 presents the changes over time in the overall PHQ9 score and each item score in the two groups. In the experimental group compared to the control group, there was a significant improvement in the total PHQ9 score (mean improvement score −2.97 ± 0.58 vs. −0.28 ± 1.06, F = 171.9; *p* < 0.00001) and in the scores of items such as the following:

N°2: Feeling down, depressed, or hopeless (mean improvement score −0.23 ± 0.12 vs. +0.08 ± 0.20, F = 60.22; *p* < 0.00001);

N°3: Trouble falling or staying asleep or sleeping too much (mean improvement score −0.48 ± 0.18 vs. –0.07 ± 0.20, F = 72.461; *p* < 0.00001);

N°5: Poor appetite or overeating (mean improvement score −0.26 ± 0.14 vs. +0.08 ± 0.17, F = 79.90; *p* < 0.00001);

N°6: Feeling bad about oneself, feeling like a failure, or feeling that one has let oneself or one’s family down (mean improvement score −0.46 ± 0.15 vs. −0.08 ± 0.25, F = 57.91; *p* < 0.00001);

N°7: Trouble concentrating on things such as reading the newspaper or watching television (mean improvement score −0.41 ± 0.15 vs. −0.04 ± 0.24, F = 57.79; *p* < 0.00001);

N°8: Being so fidgety or restless that one has been moving around a lot more than usual or moving or speaking so slowly that others might have noticed (mean improvement score −0.26 ± 0.12 vs. −0.04 ± 0.18, F = 44.92; *p* < 0.00001);

N°9: Thoughts that one would be better off dead or thoughts of hurting oneself in some way (mean improvement score −0.08 ± 0.09 vs. +0.12 ± 0.13, F = 52.959; *p* < 0.00001).

There were no statistically significant differences in the change by time in the two groups in the mean responses of the items N°1—Little interest or pleasure in doing things and N°4—Feeling tired or having little energy.

## 4. Discussion

Our study showed that the preliminary result emerging from the first article on the randomized–controlled study NCT05070065, i.e., that the virtual reality intervention lowered the depressive symptoms [21], is of such magnitude that it can have an impact on the clinical and preventive field. In fact, the data of this study showed a decrease from T0 to T1 in the experimental group (which underwent the virtual reality training) in the number of those who had a score on PHQ9 (measure of depressive state) that would be considered positive in a screening survey for a depressive episode (i.e., overall score greater than 9). The decrease was greater in comparison to the control group. As explained in the methodology, the sample of this study is characterized by people with a diagnosis of bipolar disorder in a current condition of stability without crises. However (as is typical of people who access a specialized tertiary care center), most of them show several residual depressive symptoms despite an acceptable level of social functioning. This condition is at high risk of relapse [13,33,34] and can compromise the course of treatment because it is associated with low adherence to therapy [14]; thus, having a tool that, perhaps through future improvements, can be capable of lowering the number of depressive symptoms to the point that approximately one-quarter of patients descend the risk threshold could be an important element in the field of prevention of relapses of bipolar disorder [35].

Although interesting research is ongoing about the potential use of virtual reality in major depressive disorder, with encouraging findings, most of them are pilot studies [36,37,38,39], and there is no similar literature relating to bipolar disorder. The application in the field of bipolar disorder has so far been highly experimental and suggestive of further developments, such as creating virtual environments that indicate induced excitement and tension “inducing relevant state aspects of hypomania … suitable as a paradigm for future experimental studies” [40]. The same applies to the development of experimental interventions to work on the emotional dysregulation of people with bipolar disorder and their relatives, a field that seems very promising but of which there is still a lack of data regarding the repercussions in terms of outcome because it is in early development [41,42,43]. 

From this perspective, our study serves as a significant starting point despite its primary focus on cognitive performance enhancement rather than directly targeting depressive symptoms. To comprehend the intervention’s mechanism, insights from studies investigating social cognition deficits among euthymic individuals with BD can be useful [44,45]. Social cognition encompasses both cognitive and affective aspects, with impairments in these domains correlating with residual BD symptoms [46,47]. Exploring the relationship between cognitive remediation interventions and social cognition deficits in BD individuals may shed light on the underlying mechanisms of our intervention’s efficacy [48,49]. Virtual reality (VR) technology facilitates the examination of these relationships, providing a platform to assess cognitive and emotional processing in controlled environments [27]. Understanding how cognitive stimulation interventions impact social cognition in BD individuals is crucial for optimizing treatment strategies [50]. By addressing deficits in social cognition, interventions may indirectly alleviate depressive symptoms and reduce the risk of relapse in BD patients. Furthermore, elucidating the neural correlates of social cognition deficits in BD using VR technology can inform the development of targeted interventions [51]. By targeting specific brain regions associated with social cognition impairments, such interventions may yield more significant improvements in depressive symptomatology and overall functional outcomes. Our study lays the groundwork for future research aimed at elucidating the interplay between cognitive enhancement interventions, social cognition deficits, and depressive symptoms in BD. Leveraging VR technology and insights from the existing literature can guide the development of more effective therapeutic approaches tailored to the unique needs of individuals with BD. To understand how the intervention may have worked, it may be useful to recall some recent studies that have shown (again, thanks to the use of virtual reality) that people suffering from bipolar disorder in euthymia have a serious deficit in social cognition activities [52,53,54]. Social skill involves both cognitive and affective processing, impairments of which have been found linked to residual symptoms of bipolar disorder because the implications in the impairments in processing do not change from a cognitive perspective when appropriate [53,55,56,57,58]. This impairment compromises the ability to understand the emotions and intentions of others but also may limit the understanding of their own symptoms [52,55,59,60]. The combination of virtual reality and imaging has made it possible to verify that when faced with emotional conditions that require cognitive responses, while controls without bipolar disorders activated the right anterior cingulate cortex, the insular cortex, and the inferior frontal cortex, people with bipolar disorder had reduced activations in the “mirror neuron system” (i.e., the right inferior frontal cortex, the insula, and the premotor cortex) [52,61]. This may explain why undergoing cognitive remediation training (although it would be more correct at this point to call it “cognitive stimulation”) can improve depressive symptoms. Above all, this is because the environment does not involve high-stress stimuli (because in virtual reality, the person is alone, and the clinician calibrates the tasks) and was built in such a way that the skills learned can be easily generalizable in daily life, the process of which was fun and not boring (which is essential for people with the basic characteristics of bipolar disorder). In fact, the depressive symptoms that improved the most in the experimental versus control group comparison were precisely those linked to self-esteem (N°6—“Feeling bad about yourself or that you are a failure or have disappointed yourself or your family”) and those linked to dysregulation of sleep (N°3—Difficulty falling or staying asleep or sleeping too much). This may support the hypothesis that virtual reality affects self-esteem by improving skills in emotional cognition and consequently improves stress symptoms (such as sleep and appetite dysregulation), known as important elements linking residual symptoms with triggering relapses [12,62,63] and suicidal ideation in bipolar disorders [64,65].

The intervention seems to act on key elements of functioning (social cognition, frustration, and stress with dysregulation of rhythms) that seem to be typical of “DYMERS”, i.e., a recently described stress syndrome [17,66] that, in people with a profile of hyper-energy, hyperactivity, and novelty-seeking [17,67], can represent an antecedent of bipolar disorder [67,68] and/or other disorders [18].

From this point of view, cognitive stimulation intervention could be useful not only in the field of prevention of relapses in bipolar disorder (where the positive effects could be immense if the result were reconfirmed) but also in the reduction in risk for at-risk people.

## 5. Conclusions

The findings seem to suggest that cognitive stimulation utilizing virtual reality may offer utility in the field of preventing relapses in bipolar disorder. Prevention of the relapse of depressive symptoms could indeed serve as an essential factor in enhancing the course of bipolar disorder. Cognitive stimulation seems to target the decline in social cognition and subsequent symptoms of low self-esteem and stress-related rhythm dysregulation. Moreover, the intervention holds promise for preempting relapses in individuals at risk. Further validation of our study outcomes is warranted through phase-three trials conducted in a multicenter manner. This robust approach will help solidify the reliability and generalizability of our findings, bolstering their applicability in clinical settings. Additionally, examining the long-term effects and scalability of cognitive stimulation interventions through multicenter studies can provide valuable insights into their sustained efficacy and potential integration into routine care protocols for bipolar disorder management.

## Figures and Tables

**Table 1:** Change by time in people with PHQ9 > 9 in the two groups.

	T0	T1	N	Castellan Chi-Square
PHQ9 > 9 EX	28 (71.8%)	19 (48.7%)	39	6.71
PHQ9 > 9 CC	10 (40%)	10 (40%)	25	*p* = 0.01

EX: experimental group; CC: control group.

**Table 2:** Change by time of the overall score and of each item of PHQ9 in the two groups.

	PHQ9 T0	PHQ9 T1	Mean Score
T0 PHQ9 Total (*n* = 39) EX	13.72 ± 6.12	10.82 ± 6.45	−2.90 ± 0.58
T0 PHQ9 Total (*n* = 25) CC	12.20 ± 6.26	11.92 ± 7.45	−0.28 ± 1.06
			F = 163.11; *p* < 0.00001
T0 PHQ9 Item1 (*n* = 39) EX	1.56 ± 0.92	1.20 ± 0.85	−0.36 ± 0.16
T0 PHQ9 Item1 (*n* = 25) CC	1.56 ± 0.98	1.24 ± 0.94	−0.32 ± 0.24
Little interest or pleasure in doing things			F = 0.64; *p* = 0.42
T0 PHQ9 Item 2 (*n* = 39) EX	1.56 ± 0.89	1.30 ± 0.96	−0.23 ± 0.12
T0 PHQ9 Item 2 (*n* = 25) CC	1.36 ± 0.93	1.44 ± 1.02	+0.08 ± 0.20
Feeling down, depressed, or hopeless			F = 60.22; *p* < 0.00001
T0 PHQ9 Item 3 (*n* = 39) EX	1.84 ± 1.18	1.38 ± 1.16	−0.48 ± 0.18
T0 PHQ9 Item 3 (*n* = 25) CC	1.88 ± 1.03	1.75 ± 1.19	−0.07 ± 0.20
Trouble falling or staying asleep or sleeping too much			F = 72.46; *p* < 0.00001
T0 PHQ9 Item 4 (*n* = 39) EX	1.64 ± 1.04	1.66 ± 0.91	0.02 ± 0.12
T0 PHQ9 Item 4 (*n* = 25) CC	1.56 ± 0.89	1.68 ± 1.04	0.08 ± 0.17
Feeling tired or having little energy			F = 2.74; *p* = 10.3
T0 PHQ9 Item 5 (*n* = 39) EX	1.60 ± 1.03	1.34 ± 1.00	−0.26 ± 0.14
T0 PHQ9 Item 5 (*n* = 25) CC	1.40 ± 1.13	1.48 ± 1.13	+0.08 ± 0.17
Poor appetite or overeating			F = 79.90; *p* < 0.0001
T0 PHQ9 Item 6 (*n* = 39) EX	1.56 ± 1.07	1.10 ± 0.98	−0.46 ± 0.15
T0 PHQ9 Item 6 (*n* = 25) CC	1.36 ± 1.05	1.28 ± 1.04	−0.08 ± 0.25
Feeling bad about yourself or that you are a failure or have let yourself or your family down			F = 57.91; *p* < 0.0001
T0 PHQ9 Item 7 (*n* = 39) EX	1.76 ± 1.04	1.35 ± 0.89	−0.41 ± 0.15
T0 PHQ9 Item 7 (*n* = 25) CC	1.40 ± 1.09	1.36 ± 0.97	−0.04 ± 0.24
Trouble concentrating on things such as reading the newspaper or watching television			F = 57.79; *p* < 0.0001
T0 PHQ9 Item 8 (*n* = 39) EX	1.10 ± 1.08	0.84 ± 1.07	−0.26 ± 0.12
T0 PHQ9 Item 8 (*n* = 25) CC	1.08 ± 0.89	1.04 ± 1.11	−0.04 ± 0.18
Moving or speaking so slowly that other people could have noticed or the opposite—being so fidgety or restless that you have been moving around a lot more than usual			F = 44.92; *p* < 0.0001
T0 PHQ9 Item 9 (*n* = 39) EX	0.64 ± 0.94	0.56 ± 0.81	−0.08 ± 0.09
T0 PHQ9 Item 9 (*n* = 25) CC	0.60 ± 0.89	0.72 ± 1.07	+0.12 ± 0.13
Thoughts that you would be better off dead or thoughts of hurting yourself in some way			F = 52.95; *p* < 0.0001

EX: experimental group; CC: control group.

## Data Availability

Data is contained within the article.
